# Supplementary material for: Transfer Learning in Trajectory Decoding: Sensor or Source Space?
Source: Sensors (Basel). 2023 Mar 30;23(7):3593. doi: 10.3390/s23073593 (PMC10098869; doi:10.3390/s23073593)
Supplement: Supplementary file 1 [file sensors-23-03593-s001.zip › sensors-2261644-supplementary.pdf]

Transfer learning in trajectory decoding: Sensor or source space?

Authors
Nitikorn Srisrisawang [1] and Gernot R. Müller-Putz [1, 2, *]
1 Institute of Neural Engineering, Graz University of Technology, Stremayrgasse 16/IV, 8010 Graz, Austria
2 BioTechMed Graz, Austria
* Corresponding author. Institute of Neural Engineering, Graz University of Technology, Graz, Styria, Austria. E-mail address: gernot.mueller@tugraz.at (G.R. Müller-Putz)
Keywords: electroencephalography, brain-computer interface, source localization, trajectory decoding, partial least squares regression, unscented Kalman filter, transfer learning

Supplementary materials

Table S1: The median correlations corresponding to Figure 3. The value in parentheses indicates the standard deviation.

Session	%EEG	Gen		GenC		Ind		IndC	
		Se	SS	Se	SS	Se	SS	Se	SS
1	0%	0.04 (0.03)	0.02 (0.02)	0.04 (0.03)	0.02 (0.02)	0.04 (0.03)	0.02 (0.02)	0.04 (0.03)	0.02 (0.02)
	50%	0.05 (0.06)	0.05 (0.05)	0.08 (0.05)	0.08 (0.04)	0.18 (0.08)	0.17 (0.08)	0.18 (0.08)	0.17 (0.08)
	100%	0.05 (0.04)	0.04 (0.04)	0.07 (0.06)	0.07 (0.06)	0.10 (0.07)	0.09 (0.07)	0.10 (0.07)	0.09 (0.07)
2	0%	0.07 (0.04)	0.05 (0.04)	0.08 (0.04)	0.07 (0.03)	0.09 (0.04)	0.06 (0.05)	0.09 (0.04)	0.06 (0.05)
	50%	0.09 (0.06)	0.06 (0.04)	0.11 (0.08)	0.14 (0.08)	0.19 (0.10)	0.18 (0.09)	0.16 (0.08)	0.17 (0.08)
	100%	0.06 (0.04)	0.05 (0.06)	0.09 (0.06)	0.09 (0.07)	0.15 (0.09)	0.15 (0.09)	0.14 (0.08)	0.15 (0.07)
3	0%	0.06 (0.05)	0.06 (0.04)	0.08 (0.05)	0.08 (0.04)	0.10 (0.06)	0.10 (0.05)	0.12 (0.06)	0.10 (0.05)
	50%	0.03 (0.05)	0.05 (0.04)	0.07 (0.05)	0.10 (0.05)	0.15 (0.06)	0.13 (0.05)	0.13 (0.05)	0.14 (0.04)
	100%	0.06 (0.03)	0.04 (0.02)	0.10 (0.04)	0.08 (0.05)	0.11 (0.05)	0.11 (0.05)	0.12 (0.06)	0.12 (0.05)

Table S2: The median SNRs corresponding to Figure 4. The value in parentheses indicates the standard deviation.

Session	%EEG	Gen		GenC		Ind		IndC	
		Se	SS	Se	SS	Se	SS	Se	SS
1	0%	-4.97 (1.35)	-5.45 (1.97)	-4.97 (1.35)	-5.45 (1.97)	-4.97 (1.35)	-5.45 (1.97)	-4.97 (1.35)	-5.45 (1.97)
	50%	-5.71 (1.48)	-5.85 (2.00)	-5.85 (1.58)	-5.70 (1.71)	-2.95 (1.05)	-3.11 (1.11)	-2.95 (1.05)	-3.11 (1.11)
	100%	-5.30 (1.27)	-5.35 (1.31)	-5.43 (1.37)	-5.32 (1.45)	-3.52 (1.05)	-3.53 (1.01)	-3.52 (1.05)	-3.53 (1.01)
2	0%	-5.33 (1.15)	-4.92 (1.01)	-5.32 (1.09)	-4.89 (0.95)	-3.57 (0.89)	-4.11 (1.71)	-3.57 (0.89)	-4.11 (1.71)
	50%	-5.32 (1.56)	-5.25 (1.33)	-5.64 (1.64)	-5.34 (1.35)	-2.87 (0.49)	-3.10 (0.54)	-3.24 (0.40)	-3.36 (0.38)
	100%	-5.43 (1.40)	-5.38 (1.41)	-5.74 (1.20)	-5.54 (1.38)	-2.88 (0.76)	-3.01 (0.69)	-3.28 (0.50)	-3.28 (0.58)
3	0%	-4.69 (1.74)	-4.79 (1.77)	-4.57 (1.43)	-4.77 (1.51)	-3.32 (1.16)	-3.05 (0.94)	-3.15 (0.89)	-3.33 (0.72)
	50%	-4.87 (1.45)	-5.03 (1.58)	-4.76 (1.13)	-4.80 (1.10)	-2.96 (0.86)	-3.21 (0.88)	-3.12 (0.58)	-3.15 (0.39)
	100%	-4.96 (1.44)	-5.29 (1.53)	-4.72 (0.91)	-5.13 (1.15)	-3.05 (0.72)	-3.07 (0.67)	-3.20 (0.30)	-3.08 (0.26)

Table S3: P-values obtained from pairwise two-tailed Wilcoxon signed-rank tests for the **correlations** in **position**. The color indicates the significant level: Green (<0.05), Blue (<0.01), and Purple (<0.001). P-values are adjusted to control the false discovery rate (FDR).

Se-Gen								
Se-GenC	5.2e-09							
Se-Ind	5.8e-13	9.7e-10						
Se-IndC	2e-12	1.3e-08	0.0445					
SS-Gen	0.1196	3.2e-07	2.8e-14	1.9e-13				
SS-GenC	1.3e-04	0.5972	2.55e-09	3.9e-07	1.4e-08			
SS-Ind	9.3e-10	2.1e-06	0.0035	0.5198	3.4e-11	2.8e-06		
SS-IndC	4.7e-10	3.9e-07	0.0074	0.5350	3.8e-12	2.2e-06	0.8263	
	Se-Gen	Se-GenC	Se-Ind	Se-IndC	SS-Gen	SS-GenC	SS-Ind	SS-IndC

Table S4: P-values obtained from pairwise two-tailed Wilcoxon signed-rank tests for the **correlations** in **velocity**. The color indicates the significant level: Green (<0.05), Blue (<0.01), and Purple (<0.001). P-values are adjusted to control the false discovery rate (FDR).

Se-Gen								
Se-GenC	2.6e-10							
Se-Ind	5.9e-13	2.9e-09						
Se-IndC	1.1e-12	3.3e-08	0.0643					
SS-Gen	0.0427	1e-08	2.8e-14	4.5e-14				
SS-GenC	3.4e-04	0.8527	3.2e-09	3e-07	6e-09			
SS-Ind	2.5e-09	7.3e-06	0.0013	0.3404	1.8e-11	2.8e-06		
SS-IndC	4e-10	2.1e-06	0.0038	0.4652	2.2e-12	3e-06	0.8081	
y/x	Se-Gen	Se-GenC	Se-Ind	Se-IndC	SS-Gen	SS-GenC	SS-Ind	SS-IndC

Table S5: P-values obtained from pairwise two-tailed Wilcoxon signed-rank tests for the **correlations** in **non-linear kinematics**. The color indicates the significant level: Green (<0.05), Blue (<0.01), and Purple (<0.001). P-values are adjusted to control the false discovery rate (FDR).

Se-Gen								
Se-GenC	1.2e-06							
Se-Ind	1.8e-05	0.1396						
Se-IndC	5.2e-08	9.2e-04	0.0156					
SS-Gen	0.6665	6.5e-04	8e-06	9.9e-09				
SS-GenC	0.0060	0.0882	0.0076	8.6e-06	0.0081			
SS-Ind	7.6e-05	0.1342	0.9419	0.06	5.6e-06	0.0074		
SS-IndC	1.5e-05	0.0445	0.7764	0.0277	1e-06	3.7e-04	0.5424	
y/x	Se-Gen	Se-GenC	Se-Ind	Se-IndC	SS-Gen	SS-GenC	SS-Ind	SS-IndC

Table S6: P-values obtained from pairwise two-tailed Wilcoxon signed-rank tests for the **SNRs** in **position**. The color indicates the significant level: Green (<0.05), Blue (<0.01), and Purple (<0.001). P-values are adjusted to control the false discovery rate (FDR).

Se-Gen								
Se-GenC	0.9114							
Se-Ind	1e-23	4.3e-24						
Se-IndC	3.3e-24	2.3e-24	0.0353					
SS-Gen	0.1180	0.1463	3.6e-24	3.7e-25				
SS-GenC	0.7251	0.7658	2.9e-24	3.7e-25	0.0412			
SS-Ind	5.2e-21	1.4e-21	9.5e-05	0.3710	7.4e-22	3.9e-22		
SS-IndC	3.2e-21	1.1e-21	2.2e-04	0.0108	1.6e-22	6.1e-23	0.1823	
y/x	Se-Gen	Se-GenC	Se-Ind	Se-IndC	SS-Gen	SS-GenC	SS-Ind	SS-IndC

Table S7: P-values obtained from pairwise two-tailed Wilcoxon signed-rank tests for the **SNRs** in **velocity**. The color indicates the significant level: Green (<0.05), Blue (<0.01), and Purple (<0.001). P-values are adjusted to control the false discovery rate (FDR).

Se-Gen								
Se-GenC	0.8693							
Se-Ind	6.1e-23	1.4e-23						
Se-IndC	1.3e-23	3.3e-24	0.0123					
SS-Gen	0.1196	0.1405	1.3e-23	1.2e-24				
SS-GenC	0.8081	0.7917	6.2e-24	7.6e-25	0.0707			
SS-Ind	7.5e-20	1e-20	2.6e-04	0.7764	2.4e-21	8.3e-22		
SS-IndC	3.5e-20	7.9e-21	2.7e-04	0.0659	4.5e-22	1.1e-22	0.0989	
y/x	Se-Gen	Se-GenC	Se-Ind	Se-IndC	SS-Gen	SS-GenC	SS-Ind	SS-IndC

Table S8: P-values obtained from pairwise two-tailed Wilcoxon signed-rank tests for the **SNRs** in **non-linear kinematics**. The color indicates the significant level: Green (<0.05), Blue (<0.01), and Purple (<0.001). P-values are adjusted to control the false discovery rate (FDR).

Se-Gen								
Se-GenC	0.0137							
Se-Ind	1.4e-23	4.8e-24						
Se-IndC	3.3e-24	3.7e-25	0.0343					
SS-Gen	0.4652	0.6456	3.4e-23	7.6e-24				
SS-GenC	0.4130	0.7859	3.33e-24	3.7e-25	0.1228			
SS-Ind	1.3e-19	2.4e-21	0.0230	0.8246	5.8e-21	6.6e-22		
SS-IndC	1e-19	1.3e-21	0.0012	0.0213	1.7e-21	3.1e-23	0.0165	
y/x	Se-Gen	Se-GenC	Se-Ind	Se-IndC	SS-Gen	SS-GenC	SS-Ind	SS-IndC

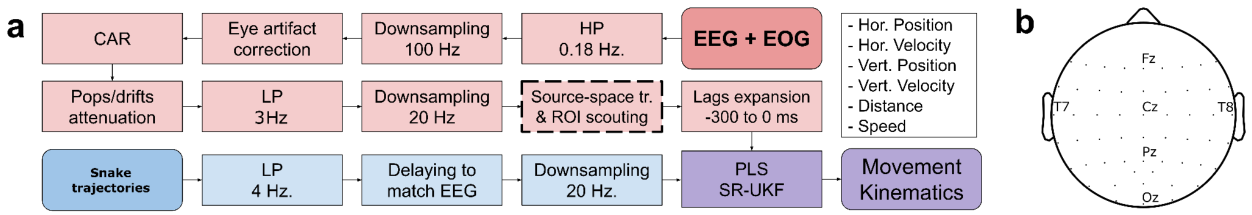

Figure S1: a) Signal processing pipeline of the EEG, EOG signals and the corresponding snake trajectories. b) Electrodes layout for the EEG signals

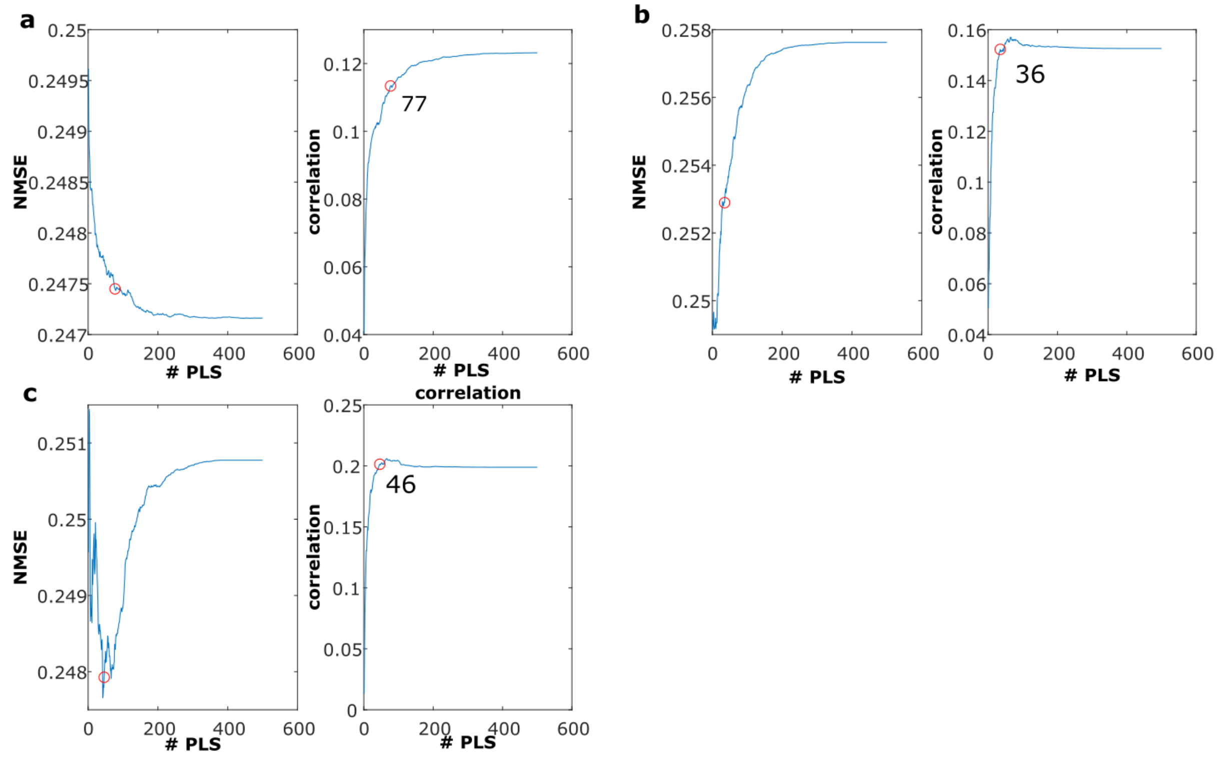

Figure S2: Exemplary of plots between normalized mean squares error (NMSE) and correlation between PLS predicted kinematics (only position and velocity) and the ground truth kinematics with a different number of PLS latent components. The NMSE and correlation shown are the average value across 4 kinematics and 10-fold cross-validation. The red circle represents the “knee” point chosen as the optimum point, and the number represents the optimum number of PLS latent components. a) the case where correlation increases while NMSE decreases, b) the case where NMSE and correlation both increase, and c) the mixed case where NMSE initially decreases but then increases again.

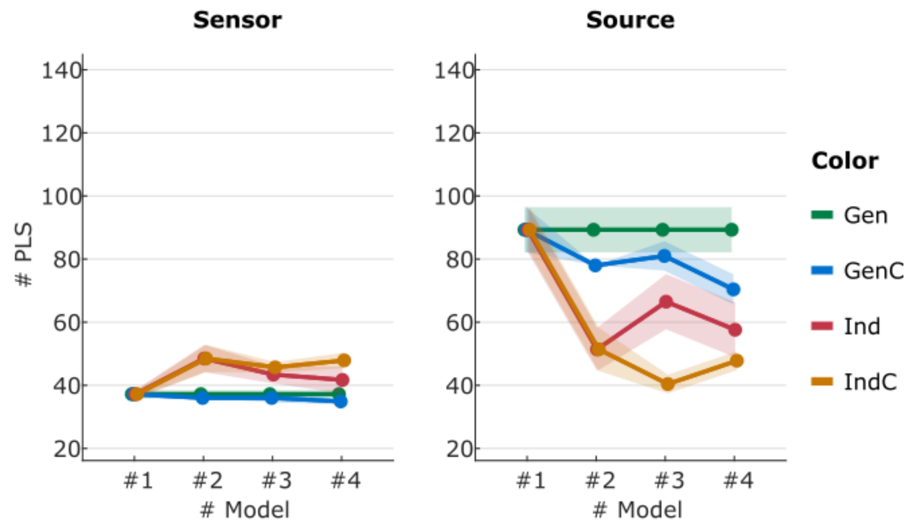

Figure S3: Average number of optimum PLS latent components of each adaptation strategy, represented by color, at each adaptation stage (see Figure 1). The shaded area represents the standard error.

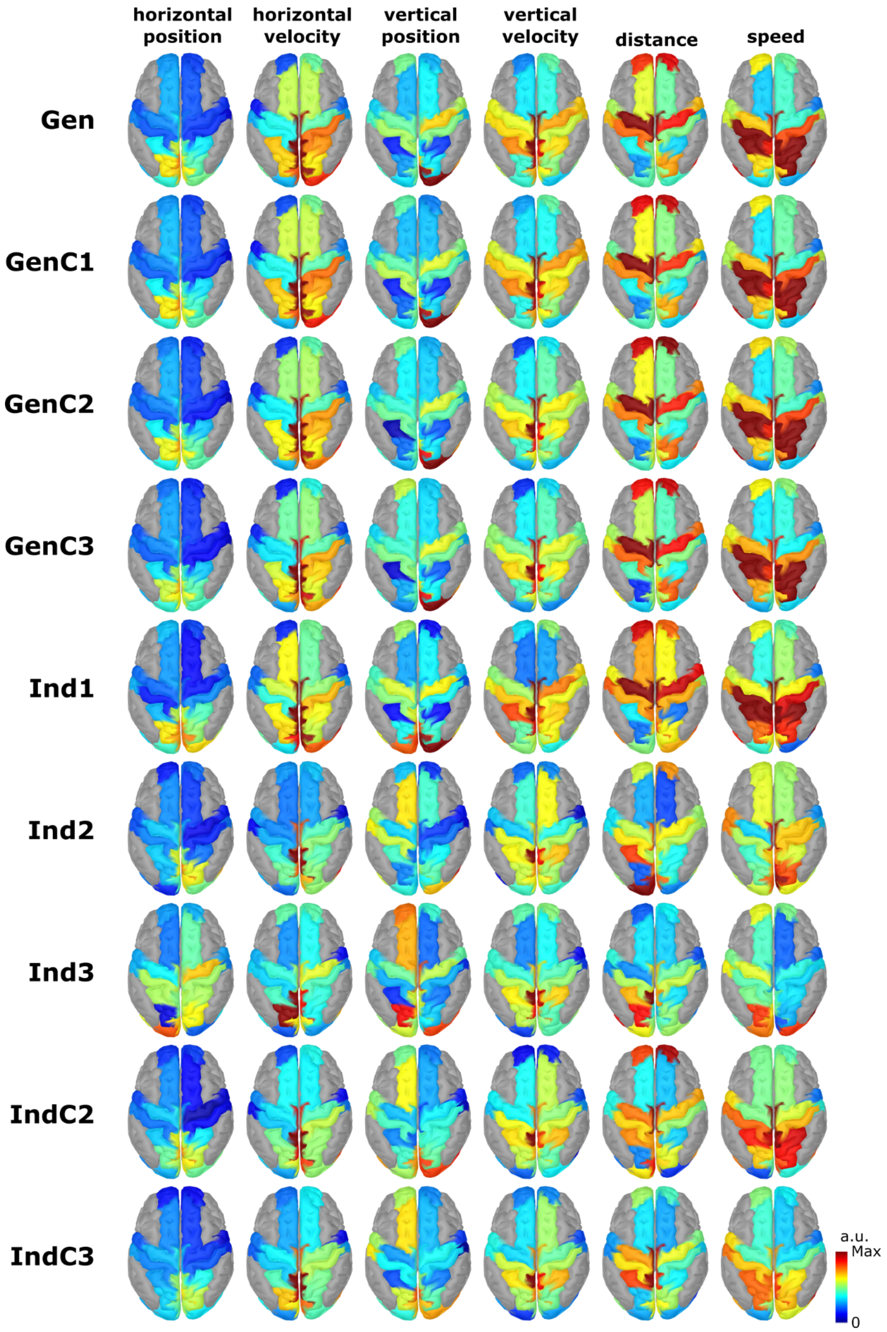

Figure S4: The group-level decoding pattern at lag 0 of all models (The naming was according to Figure 1). Blue and red indicate the value at 0 and maximum
